# Microbiome and metabolome associated with white spot lesions in patients treated with clear aligners

**DOI:** 10.3389/fcimb.2023.1119616

**Published:** 2023-04-04

**Authors:** Zhixin Song, Shishu Fang, Tao Guo, Yi Wen, Qian Liu, Zuolin Jin

**Affiliations:** ^1^ State Key Laboratory of Military Stomatology and National Clinical Research Center for Oral Diseases and Shaanxi Clinical Research Center for Oral Diseases, Department of Orthodontics, School of Stomatology, Air Force Medical University, Xi’an, China; ^2^ Department of Stomatology, General Hospital of Southern Theater Command of the Chinese People’s Liberation Army, Guangzhou, China; ^3^ Department of Orthodontics, TaiKang Shanghai Bybo Dental Hospital, Shanghai, China

**Keywords:** clear aligners, white spot lesions, adolescents, microbiota, metabolites, saliva

## Abstract

White spot lesions (WSLs) have long been a noteworthy complication during orthodontic treatment. Recently, an increasing number of orthodontists have found that adolescents undergoing orthodontic treatment with clear aligners are at a higher risk of developing WSLs. The oral microbiota and metabolites are considered the etiologic and regulatory factors of WSLs, but the specific impact of clear aligners on the oral microbiota and metabolites is unknown. This study investigated the differences in the salivary microbiome and metabolome between adolescents with and without WSLs treated with clear aligners. Fifty-five adolescents (aged 11-18) with Invisalign appliances, 27 with and 28 without WSLs, were included. Saliva samples were analyzed using 16S rRNA gene sequencing and ultra-performance liquid chromatography-tandem mass spectrometry (UPLC-MS/MS); the data were further integrated for Spearman correlation analysis. The relative abundances of 14 taxa, including *Actinobacteria*, *Actinomycetales*, *Rothia*, *Micrococcaceae*, *Subdoligranulum*, *Capnocytophaga*, *Azospira*, *Olsenella*, *Lachnoanaerobaculum*, and *Abiotrophia*, were significantly higher in the WSL group than in the control group. Metabolomic analysis identified 27 potential biomarkers, and most were amino acids, including proline and glycine. The metabolites were implicated in 6 metabolic pathways, including alanine, aspartate and glutamate metabolism; glycine, serine and threonine metabolism; and aminoacyl-tRNA biosynthesis. There was a correlation between the salivary microbial and metabolomic datasets, reflecting the impact of clear aligners on the metabolic activity of the oral flora. A concordant increase in the levels of *Lachnoanaerobaculum*, *Rothia*, *Subdoligranulum* and some amino acids had predictive value for WSL development. In summary, when adolescents undergo long-term clear aligner therapy with poor oral hygiene habits, clear aligners can disrupt the balance of the oral microecosystem and lead to oral microbiota dysbiosis, thereby increasing the risk of developing WSLs. Our findings might contribute to the understanding of the pathogenesis of WSLs and provide candidate biomarkers for the diagnosis and treatment of WSLs associated with clear aligners.

## Introduction

With the development of biomechanics and 3D technology, clear aligners have been widely applied in malocclusion treatments and are also popular among adolescents (Alansari, 2020). Previous studies have shown that orthodontic treatment with fixed appliances carries a risk of complications, among which white spot lesions (WSLs) are one of the most common adverse effects that can have lasting negative impacts on dental esthetics and can progress to severe caries, leading to the destruction of multiple dental tissues (Kozak et al., 2020). The implantation of orthodontic devices and poor oral hygiene in the oral cavity can cause the accumulation of plaque biofilms and change the oral microecological balance, thus causing or aggravating WSLs, periodontal disease and other oral diseases (Ong and Wang, 2002). Unlike fixed orthodontic appliances, clear aligners can be removed, making oral hygiene maintenance easier (Rossini et al., 2015). Recent studies have suggested that it is easy for patients treated with clear aligners to perform oral cleaning and maintain periodontal health and that these patients may have a lower risk of developing WSLs than those treated with fixed orthodontic appliances (Buschang et al., 2019); however, in clinical practice, we have found that the incidence of WSLs after wearing clear aligners is not low in adolescents in Xi’an, China, at approximately 34.6%, probably because of the significant differences in oral hygiene practices, diet and hormone levels in adolescents compared to adults. In addition, clear aligners cover the entire tooth surface for a long time, which limits the self-cleaning effect of saliva (Bisht et al., 2022), and the oral microenvironment of adolescents may change, which is more likely to cause enamel demineralization. However, the etiology of this phenomenon has not been studied.

WSLs are an early manifestation of caries caused by an imbalance between demineralization and remineralization of tooth enamel, which depends on the balance between oral microorganisms, availability of fermentable carbohydrates and salivary protective factors (Moslemi et al., 2015). Previously, a large number of studies have reported pathogenic bacteria related to caries, among which *Streptococcus mutans* and *Lactobacillus* species are recognized as cariogenic bacteria, and their acid production and acid resistance properties are closely related to caries. In addition, a few studies have reported the pathogenic bacteria related to WSLs. Jiang et al. found that *Actinomyces* and *Corynebacterium* were involved in the initial stage of caries and were related to the formation of WSLs (Jiang et al., 2014). Tanner et al. reported that *Granulicatella*, *Veillonellaceae*, *Bifidobacteriaceae*, *Streptococcus mutans*, and *Scardovia wiggsiae* were associated with the presence of WSLs in adolescents undergoing fixed orthodontic treatment (Tanner et al., 2012). However, most previous studies have used bacterial culture or PCR techniques to study several caries-causing bacteria (Al-Melh et al., 2020). With the advent of high-throughput sequencing technology, recent studies have indicated that WSLs might be caused by multiple microorganisms (Havsed et al., 2021). Given that saliva can be collected noninvasively and is in contact with multiple ecological niches within the oral cavity, a comprehensive analysis of the microbial community in saliva has been proposed to better explain the etiology of WSLs (Li et al., 2021). The oral microbiota is capable of converting complex chemicals in saliva into small-molecule metabolites. These metabolites can be used to assess the caries-causing potential of the oral microbiota and to establish the connection between the oral microbiota and diseases (Takahashi, 2015). However, the impact of interactions between the microbiota and metabolites on the development of WSLs caused by clear aligners has not been elucidated thus far.

The aim of the present study was to investigate the possible pathogenesis of WSLs in adolescents undergoing orthodontic treatment with clear aligners and compare the colonization patterns of the salivary microbiota in adolescents with and without WSLs who were treated with clear aligners. We also identified changes in salivary metabolites in adolescents with WSLs and explored the correlation between the salivary microbiota and metabolites.

## Materials and methods

### Study population and sample collection

This study was approved by the School of Stomatology, Air Force Medical University Ethical Review Board, Xi’an, China. Informed written consent was obtained from the legal parents or other guardians of all subjects prior to the start of the study. This study was a cross-sectional study and was conducted in accordance with the STROBE guidelines. Adolescents (n=205) with WSLs (n=81) and those without WSLs (n=124) who were treated with clear aligners in the Department of Orthodontics, School of Stomatology, Air Force Medical University, Xi’an, China, from October 2019 to October 2021 were recruited. Patients were selected based on the following inclusion criteria: they were aged 11-18 years, had permanent dentition, changed their clear aligners (Invisalign^®^) every 7-10 days, and had been treated with clear aligners for more than one year. Invisalign patients with periodontal diseases, generalized caries, systemic diseases, or antibiotic treatment in the previous 3 months were excluded. Twenty-seven WSL patients (WSL group) and twenty-eight non-WSL patients (control group) were randomly selected for microbial and metabolomic analysis.

The detection of WSLs was performed by two Chinese orthodontists on computer monitors in a darkroom. They independently evaluated WSLs on intraoral photographs of the patients before and during treatment and had to agree on the assessments. Patients in the WSL group had at least one tooth with a WSL. Any new white spot that formed during treatment was counted as a WSL. If the same white spot was present on the photographs before and during treatment, it was not recorded as a WSL; if it worsened during treatment, it was considered a WSL. Information about the general sociodemographic characteristics, oral hygiene habits, and dietary habits of individuals in the two groups was obtained by questionnaire. Samples were collected from 9:30-10:00 am to minimize disturbance of circadian rhythms. Subjects were instructed to avoid drinking or eating 1 hour prior to saliva collection and to rest for at least 20 minutes prior to sample collection. Subjects were instructed to sit in a comfortable position with their heads slightly tilted forward to allow saliva to gradually flow into sterile centrifuge tubes, and 5 ml of non-stimulated whole saliva samples was collected from each participant. The collected samples were temporarily stored in liquid nitrogen and then in a −80°C freezer (Jiang et al., 2018). The clinical phase of the study was conducted between October 2020 and October 2021.

### 16S rRNA gene sequencing and data analysis

Microbial DNA was extracted from the samples using the Omega Mag-bind soil DNA kit (Norcross, GA, USA). The concentration and quality of DNA were assessed by a NanoDrop 2000 spectrophotometer (Thermo Scientific, USA) and agarose gel electrophoresis. The microbial 16S rRNA (V3-V4) genes were amplified with the primers 338F-806R. Amplicons were then purified by Vazyme VAHTSTM DNA Clean Beads (Vazyme, Nanjing, China) and quantified using the Quant-iT PicoGreen dsDNA Assay Kit (Invitrogen, Carlsbad, CA, USA).

Paired-end Illumina NovaSeq reads were quality filtered, denoised, and merged, and chimeras were removed from amplicon sequence variants (ASVs) using DADA2 *via* QIIME2 version 2019.4 (Callahan et al., 2016). A phylogeny was constructed for ASVs using FastTree2 (Price et al., 2009). Taxonomy was assigned to ASVs according to Greengenes database (https://greengenes.secondgenome.com/). The alpha diversity of the samples was assessed based on the Chao1, Observed species, Simpson and Shannon indices. Comparisons of alpha diversity between the two groups were performed with the Wilcoxon rank sum test. The beta diversity of the samples was assessed by principal coordinate analysis (PCoA) based on the unweighted UniFrac and Bray-Curtis distances. Permutational multivariate analysis of variance (PERMANOVA) was used to test for differences in community structure. The differences in taxa abundances at the ASV levels were compared by MetagenomeSeq. Linear discriminant analysis (LDA) effect size (LEfSe) was used to identify microbial biomarkers with a logarithmic LDA score threshold of >2 and P<0.05 (Segata et al., 2011).

### Targeted metabolomics using UPLC-MS/MS

Targeted metabolomic analysis of saliva samples was performed by Metabo-Profile Biotechnology (Shanghai, China) according to the methodology described in previous studies (Xie et al., 2021). In brief, the samples were thawed at 4°C for 2 hours, and 100 μL of saliva was added to a 96-well plate. The samples were then lyophilized and reconstituted with 20 μL of 50% methanol. The plate was transferred to the Eppendorf epMotion workstation (Eppendorf Inc., Hamburg, Germany), and 120 μL ice-cold methanol with partial internal standards was automatically added to each sample and vortexed vigorously for 5 min. The plate was centrifuged at 4000 × g for 30 min and returned to the workstation. Freshly prepared derivative reagents (20 μL) were added to each well. The plate was sealed, and the derivatization was carried out at 30°C for 60 min. After derivatization, 330 μL of ice-cold 50% methanol solution was added to dilute the sample, followed by centrifugation at 4000 × g at 4°C for 30 min. A total of 135 μL of supernatant was transferred to a new 96-well plate with 10 μL internal standards in each well. Serial dilutions of derivatized stock standards were added to the wells. Finally, the plate was sealed for analysis.

An ultra-performance liquid chromatography-tandem mass spectrometry (UPLC-MS/MS) system (ACQUITY UPLC-Xevo TQ-S, Waters Corp., Milford, MA, USA) was used to quantitate the levels of all targeted metabolites in this study. We used UPLC columns, including an ACQUITY HPLC BEH C18 VanGuard precolumn (1.7 µm, 2.1 × 5 mm) and an ACQUITY HPLC BEH C18 analytical column (1.7 µm, 2.1 × 100 mm), for chromatographic separation of saliva samples. The column temperature was kept at 40 °C, and the injection volume of the sample was 5.0 µL. The mobile phase consisting of solvent A, 0.1% formic acid in water, and solvent B, acetonitrile/IPA (70:30), was delivered at a flow rate of 0.4 ml/min. The gradient elution was as follows: 0-1 min (5% B), 1-11 min (5-78% B), 11-13.5 min (78-95% B), 13.5-14 min (95-100% B), 14-16 min (100% B), 16-16.1 min (100-5% B), and 16.1-18 min (5% B). The mass spectrometer settings were as follows: capillary: 1.5 (ESI+), 2.0 (ESI-) Kv; source temperature: 150°C; desolvation temperature: 550°C; and desolvation gas flow: 1000 L/h.

For data processing, the raw data files generated by UPLC-MS/MS were processed using TMBQ software (v1.0, Human Metabolomics Institute, Shenzhen, Guangdong, China) to calculate the concentration of each analyte in the samples. The self-developed platform iMAP (v1.0, Metabo-Profile, Shanghai, China) was used for statistical analyses, including orthogonal partial least square discriminant analysis (OPLS-DA), univariate analysis and pathway analysis. The variable importance in the projection (VIP) was obtained based on the OPLS-DA model. Potential biomarkers of differentially expressed metabolites were identified using VIP > 1 and p < 0.05 in the Student’s t test or Wilcoxon test results based on whether the data were normally distributed. Potential biomarkers were screened through MetaboAnalyst 4.0 (http://www.metaboanalyst.ca/) to identify potential metabolic pathways. Additionally, Spearman’s rank correlation analyses were conducted to explore the correlation between metabolites and microbiota in saliva samples.

## Results

### Characteristics of the study population

Based on the study criteria, two subjects were excluded, and a total of 55 subjects were identified. The clinical demographics of the subjects and questionnaire results are presented in **Table 1**. The mean age of the subjects was 13.4 ± 2.0 years. Overall, adolescents in the WSL group had poorer oral hygiene habits than those in the control group. There were significant differences in the frequency of tooth brushing between the two groups (p<0.05). Additionally, compared to adolescents without WSLs, those with WSLs associated with clear aligners preferred to eat while wearing clear aligners and drink carbonated soft drinks.

**Table 1:** Demographics and Behavioral Characteristics of the Study Participants.

Variable	WSL group (N=27)	Control group (N=28)	p value
Age (y)	12.9 ± 1.7	13.9 ± 2.1	0.143^a^
Sex			0.036^b*^
Male	20(74.1)	13(46.4)	
Female	7(25.9)	15(53.6)	
Tooth brushing frequency			0.011^b*^
not daily	8(29.6)	2(7.1)	
1~2times/d	14(51.9)	11(39.3)	
≥3times/d	5(18.5)	15(53.6)	
Brushing duration			0.090^b^
≤2 min	22(81.5)	17(60.7)	
>2 min	5(18.5)	11(39.3)	
Use of fluoridated toothpaste			0.112^b^
Yes	3(11.1)	11(39.3)	
No	4(14.8)	4(14.3)	
Unknown	20(74.1)	13(46.4)	
Use of orthodontic toothbrush			0.478^b^
Yes	3(11.1)	5(17.9)	
No	24(88.9)	23(82.1)	
Use of mouthwash			0.245^b^
Yes	2(7.4)	5(17.9)	
No	25(92.6)	23(82.1)	
Use of dental floss			0.317^b^
Yes	1(3.7)	3(10.7)	
No	26(96.3)	25(89.3)	
Carbonated soft drink preference			0.072^b^
Do not prefer	2(7.4)	8(28.6)	
Prefer occasionally (weekly)	11(40.7)	12(42.9)	
Prefer frequently (daily)	14(51.9)	8(28.6)	
Frequency of eating while wearing clear aligners			0.038^b*^
Never	4(14.8)	12(42.9)	
Occasionally (weekly)	10(37.0)	10(35.7)	
Frequently (daily)	13(48.1)	6(21.4)	

Values are presented as the mean ± SD or n (%).

a T test.

b Chi-square analysis.

^*^P ≤ 0.05.

### Profiling of salivary microbiome

Following quality filtering, 1,689,435 total DNA sequence reads were recovered from the 55 samples, with an average of 30,717 sequence reads per sample. Of these, more than 1.6 million high-quality DNA sequence reads and 12,938 unique amplicon sequence variants were identified.

**Figure 1:**
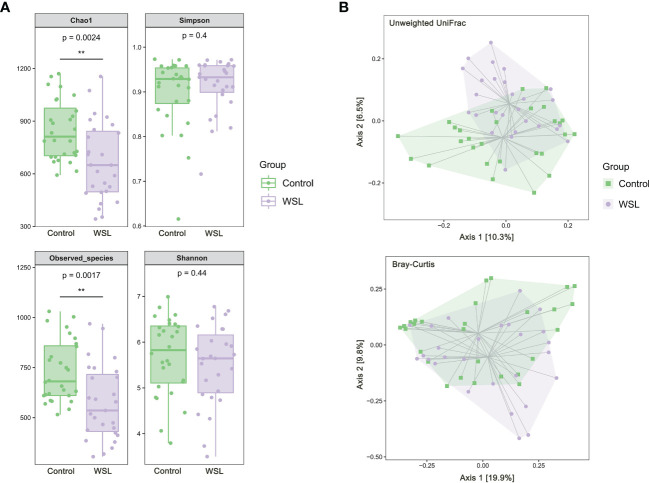
Microbial diversity analysis. **(A)** Box plots of alpha diversity of salivary microbiota in the control and WSL groups. Left: Chao1 and Observed_species indices; right: Shannon and Simpson indices (**p < 0.01, Kruskal-Wallis). **(B)** PCoA plot based on the unweighted UniFrac and Bray-Curtis distances. A certain trend of clustering and separation of samples was identified when comparing the control and WSL groups (p < 0.05, PERMANOVA).

The results of alpha diversity analysis showed that the Chao1 and Observed species indices in the WSL group were significantly lower than those in the control group (P<0.01), and the Simpson and Shannon indices were not significantly different between the two groups (**Figure 1A**). PCoA, based on unweighted UniFrac and Bray-Curtis distances, showed that although the samples of the WSL and control groups overlapped, there was a certain trend of separation between the two groups (**Figure 1B**).

**Figure 2:**
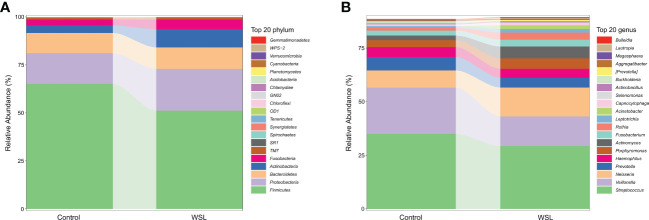
Average compositions and the relative abundance of the bacterial community in the control and WSL groups at two distinct levels. **(A)** Phylum level; **(B)** genus level.

According to the microbiome annotation results, the percentage compositions of the top 20 predominant phyla and genera in the WSL and control groups were compared to further identify the changes in microbial community structure. At the phylum level, regardless of the presence of WSLs during clear aligner therapy, the predominant phyla of all samples from both groups were *Firmicutes*, *Proteobacteria*, *Bacteroidetes*, *Actinobacteria*, *Fusobacteria* and *Candidate division TM7* (**Figure 2A**). At the genus level, genera with an average relative abundance of more than 1% were counted, and genera with high detection rates in both groups included *Streptococcus* (29.28% versus 34.92%), *Veillonella* (13.84% versus 21.65%), *Neisseria* (13.46% versus 8.00%), *Prevotella* (4.51% versus 6.26%), *Haemophilus* (4.24% versus 4.64%), *Porphyromonas* (4.86% versus 3.23%), *Actinomyces* (5.52% versus 2.14%), *Fusobacterium* (3.19% versus 2.34%), and *Rothia* (3.30% versus 1.32%), which together constituted approximately 80% of the salivary microbial community (**Figure 2B**).

**Figure 3:**
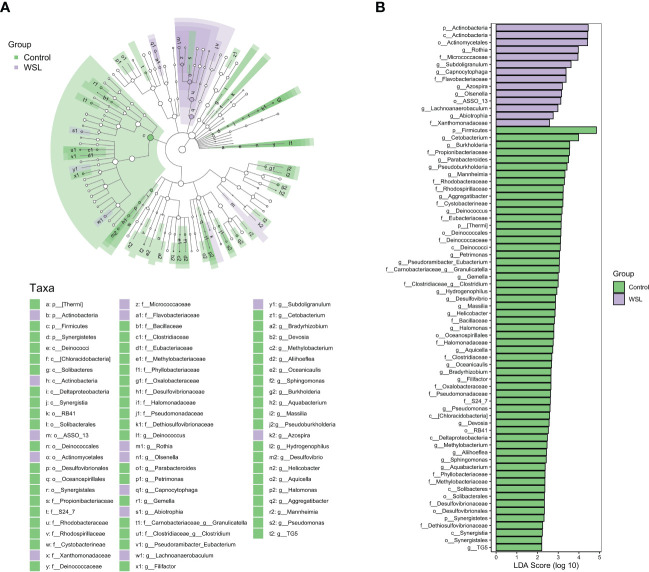
LEfSe analysis. **(A)** Cladogram showing the phylogenetic distribution of the microbiota in the control and WSL groups. The nodes radiating from inside to outside represent the taxonomic rank of the flora from phylum to genus, respectively (p, phylum; c, class; o, order; f, family; g, genus). The size of the nodes corresponds to the average relative abundance of the taxon. **(B)** Histogram of the LDA scores of taxa with different abundances between the two groups. Each bar represents the log 10 effect size (LDA score) for a specific taxon, and a longer bar represents a higher LDA score. The threshold on the logarithmic LDA score was set at 2.0.

To further characterize the changes in taxonomic composition in the two groups, we used LEfSe to generate a cladogram (**Figure 3**). The analysis results revealed distinct taxa in the oral microbiome of individuals with WSLs and those without WSLs. There were 14 taxa with higher abundances in the WSL group than in the control group, including *Actinobacteria* (at both the phylum and class levels), *Actinomycetales*, *Rothia*, *Micrococcaceae*, *Subdoligranulum*, *Capnocytophaga*, *Flavobacteriaceae*, *Azospira*, *Olsenella*, *ASSO_13*, *Lachnoanaerobaculum*, *Abiotrophia* and *Xanthomonadaceae*. In contrast, 58 taxa, including *Firmicutes*, *Cetobacterium* and *Burkholderia*, were significantly enriched in the control group.

### Profiling of salivary metabolome

**Figure 4:**
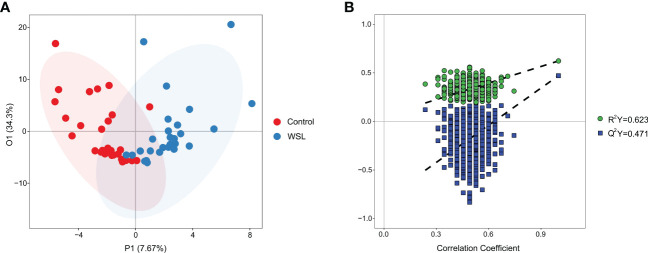
OPLS-DA. **(A)** Supervised OPLS-DA score plot of metabolite profiles in the control and WSL groups. The abscissa P1 indicates the predicted principal component score, and the ordinate O1 indicates the orthogonal principal component score. **(B)** Permutation test of the OPLS-DA model for the control and WSL groups. R^2^Y (cum) and Q^2^Y (cum) represent the interpretability and predictability of the models, respectively. Y-axis intercepts: R^2^Y=0.623, Q^2^Y=0.471.

To understand the pathogenesis and the biochemical basis underlying WSLs associated with clear aligners, we used a targeted metabolomic profiling approach to assess metabolites in the saliva. The metabolomics analysis yielded a total of 133 metabolites. The OPLS-DA model showed significant separation between the WSL and control groups, indicating a different metabolic profile in the two groups (**Figure 4A**). Permutation tests (n=1000) were performed to validate the model, and the results showed that the model was not overfitted (**Figure 4B**).

**Figure 5:**
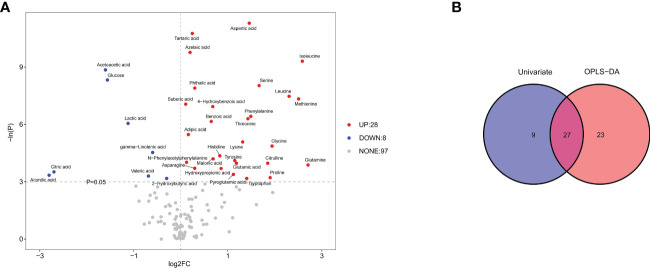
**(A)** Volcano plot of univariate statistics across the control and WSL groups. In the volcano plot, 36 differentially expressed metabolites (red and blue) and 97 nondifferentially expressed metabolites (gray) were determined under the conditions of fold change ≥ 0 and p ≤ 0.05. The red and blue dots indicate upregulated and downregulated metabolites, respectively. **(B)** Venn diagrams of differentially expressed metabolites screened by multivariate (OPLS-DA) and univariate analyses. Univariate analysis was performed by Student’s t test or the Wilcoxon test based on whether the data were normally distributed.

**Figure 6:**
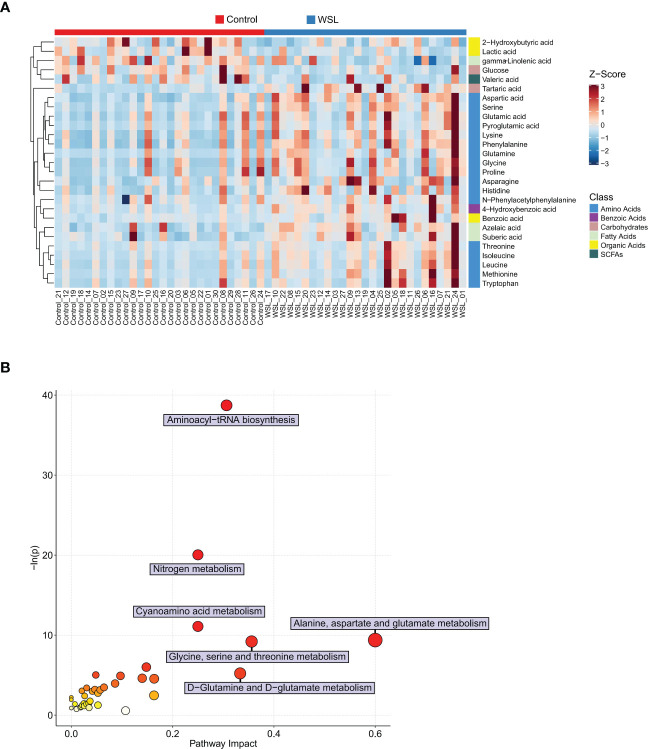
Potential biomarkers of the control and WSL groups. **(A)** Heatmap visualization of Z scores in each group. Each column represents an individual sample, and each row represents a metabolite. The color of each square represents the relative concentration of the corresponding metabolite of the sample, with red indicating a higher concentration and blue indicating a lower concentration (p < 0.05). **(B)** Bubble diagram of pathway enrichment analysis of differentially expressed metabolites. The color and size of each circle represent the p value (the redder the color, the smaller the p value) and the pathway impact value (the larger the size of the point, the higher the impact score), respectively. Pathways were considered significantly enriched if p < 0.05 and impact > 0.

Univariate statistical analysis showed that 36 metabolites, mainly amino acids (52.8%), were significantly altered in the WSL group compared to the control group (**Figure 5A**). Based on the intersection of multivariate and univariate analyses, 27 differentially expressed metabolites were chosen as potential biomarkers for the two groups (**Figure 5B**). Specifically, the levels of 22 of the 27 potential biomarkers, namely, asparagine, aspartic acid, glutamic acid, glutamine, glycine, histidine, isoleucine, leucine, lysine, methionine, N-phenylacetyl phenylalanine, phenylalanine, proline, pyroglutamic acid, serine, threonine, tryptophan, 4-hydroxybenzoic acid, tartaric acid, azelaic acid, suberic acid, and benzoic acid, were remarkably elevated in the WSL group (p<0.05). In contrast, there was a notable decrease in the relative concentration of 5 metabolites, namely, lactic acid, gamma-linolenic acid, 2-hydroxybutyric acid, valeric acid and glucose, in the WSL group. The trends in the variation in the levels of these potential biomarkers are depicted by the heatmap in **Figure 6A**.

Pathway enrichment analyses were performed to examine the impact of clear aligners on the oral metabolome. Seventeen metabolic pathways that may be associated with the development of WSLs during clear aligner therapy were identified, and the differential expressed metabolites were mainly those involved in amino acid metabolic pathways (**Figure 6B**). The following six metabolic pathways were highly enriched (impact ≥ 0.20): alanine, aspartate and glutamate metabolism; glycine, serine and threonine metabolism; aminoacyl-tRNA biosynthesis; nitrogen metabolism; cyanoamino acid metabolism; and D-glutamine and D-glutamate metabolism.

### Correlation between salivary microbiome and metabolome

**Figure 7:**
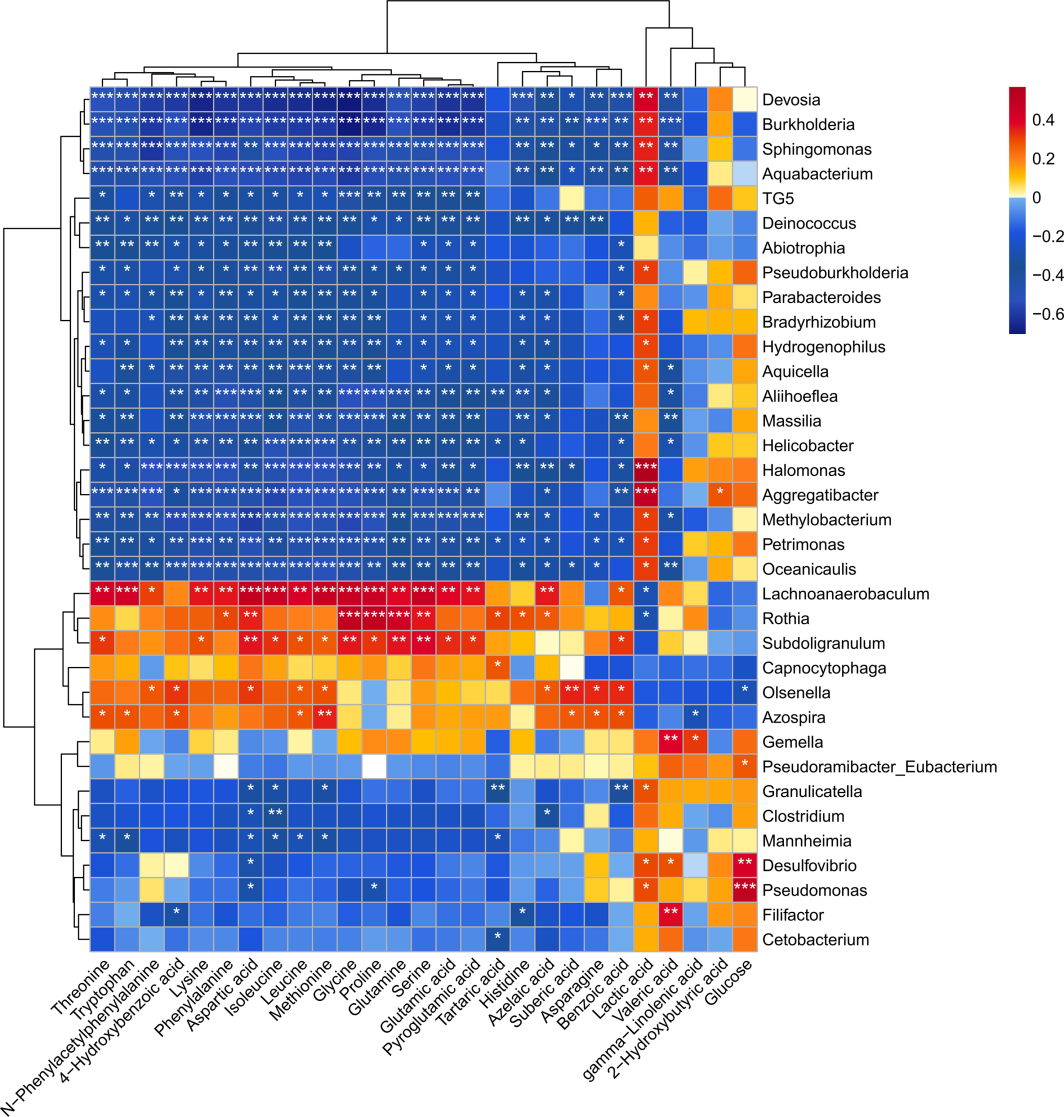
Heatmap of Spearman’s correlation coefficients between the levels of salivary microbial genera and metabolites. Each row represents a differentially expressed genus and each column represents a differentially expressed metabolite. The color of each square represents the degree of association between the genera and the metabolite, with red indicating a positive correlation and blue indicating a negative correlation. Asterisks indicate statistical significance based on the Spearman correlation. *p < 0.05, **p < 0.01, ***p < 0.001.

A heatmap was generated using data from Spearman’s correlation analysis, which allowed us to identify relationships between the disease-associated taxa and metabolites (**Figure 7**). The heatmap showed a cluster of several amino acids (glycine, proline, glutamine, serine) that were positively correlated with disease-associated microorganisms (*Lachnoanaerobaculum*, *Rothia*, *Subdoligranulum*). Specifically, *Lachnoanaerobaculum* was positively correlated with 17 metabolites, namely, tryptophan (r=0.4320), aspartic acid (r=0.4865), isoleucine (r=0.4611), methionine (r=0.4766), glycine (r=0.4662), proline (r=0.4611), serine (r=0.4662), threonine (r=0.4216), lysine (r=0.3607), phenylalanine (r=0.3611), leucine (r=0.4051), glutamine (r=0.3785), glutamic acid (r=0.3808), pyroglutamic acid (r=0.3668), azelaic acid (r=0.3569), N-phenylacetyl phenylalanine (r=0.3088) and benzoic acid (r=0.2811), and negatively correlated with lactic acid (r=-0.2816). *Rothia* was positively associated with 9 metabolites, namely, glycine (r=0.4987), proline (r=0.4968), glutamine (r=0.4417), aspartic acid (r=0.3473), serine (r=0.3603), phenylalanine (r=0.3030), tartaric acid (r=0.3072), histidine (r=0.2990) and azelaic acid (r=0.2708), and negatively associated with lactic acid (r=-0.2890). Moreover, *Subdoligranulum* was positively associated with 13 metabolites, namely, aspartic acid (r=0.3625), glycine (r=0.3597), glutamine (r=0.3631), serine (r=0.4003), threonine (r=0.3189), lysine (r=0.2911), isoleucine (r=0.3291), leucine (r=0.2869), methionine (r=0.2688), proline (r=0.3224), glutamic acid (r=0.3372), pyroglutamic acid (r=0.3242) and benzoic acid (r=0.3228). All of these correlation coefficients were statistically significant (p<0.05).

## Discussion

Many studies have demonstrated that fixed orthodontic appliances increase the risk for developing WSLs, and in most cases, they are associated with dental plaque accumulation in the region of the tooth surface around brackets (Höchli et al., 2017). However, clear aligners are made up of thermoplastic materials, which are “sleeve-like” intraoral devices that cover nearly the entire tooth surface and part of the keratinized gingiva (Zhao et al., 2020). Therefore, long-term use of clear aligners may reduce the self-cleaning ability of the tooth surface, limiting the flushing and cleaning effects of the saliva and tongue on the tooth surface and providing conditions for pathogenic bacterial proliferation, thus destroying the overall oral flora structure. Currently, little is known about how clear aligners affect the oral microbiota, and their role in the development of WSLs remains unclear. In the present study, we found that male sex, tooth brushing frequency, and eating while wearing clear aligners were risk factors for WSLs in adolescents treated with clear aligners. We also performed microbiota and targeted metabolomic profiling of saliva from 55 adolescents and identified bacterial taxa and metabolites associated with WSLs. To our knowledge, this is the first study exploring the etiology of WSLs associated with clear aligner therapy.

In the present study, both alpha diversity and beta diversity analyses showed that wearing clear aligners had an effect on the salivary microbial structure of adolescent patients. In the alpha diversity analysis, the Chao1 and Observed species indices reflected phylotype richness, and the Shannon and Simpson indices were used as indicators of diversity combining richness and community evenness (Haegeman et al., 2013). The Chao1 and Observed species indices of the WSL group in this study were both significantly lower than those of the control group, while the Shannon and Simpson indices of the two groups were not significantly different, suggesting that although wearing clear aligners has no significant impact on the diversity of the saliva microbiome, it may result in a significant reduction in the richness of the saliva microbiota in adolescent patients. This is different from previous studies on salivary microorganisms in dental caries, in which Jiang et al. found no significant difference in the richness and diversity of salivary microorganisms between patients with caries and healthy patients (Jiang et al., 2016), whereas the richness of salivary microbiota was significantly reduced in patients wearing clear aligners in this study, suggesting that clear aligners may have an effect on the salivary microbial community. In addition, the results of the beta diversity analysis in this study showed a trend of separation between the two groups of samples, indicating that the composition and structure of the salivary microbiome differed between the two groups.

Based on the results of the salivary microbiome analysis, we found that the abundance of *Actinobacteria*, *Actinomycetales*, *Rothia*, *Micrococcaceae*, *Subdoligranulum*, *Capnocytophaga*, *Azospira*, *Oisenella*, *Lachnoanaerobaculum*, and *Abiotrophia* in the saliva of patients in the WSL group was significantly higher than that in the control group. We focused on *Rothia*, *Subdoligranulum*, *Capnocytophaga*, *Olsenella*, *Lachnoanaerobaculum*, and *Abiotrophia*. First, we noticed that *Actinobacteria* (at both the phylum and class levels) and *Actinomycetales* were notably enriched in saliva samples from WSL adolescents. The bacterial genus *Actinomyces* is a common inhabitant of the oral cavity of adolescents and is closely associated with dental demineralization due to its ability to produce lactic acid under anaerobic conditions (Jiang et al., 2014). Intriguingly, the changes in *Actinobacteria* in our study only showed significant differences at the phylum, class, and order levels but not the genus level when comparing the two groups, which might be caused by the difference in sampling sites. The elevated level of *Rothia* in the WSL group also caught our attention. *Rothia*, which belongs to the family *Micrococcaceae* in the order *Actinomycetales*, was identified as one of the dominant genera in enamel carious lesions (Simón-Soro et al., 2014). Jagathrakshakan et al. discovered *Rothia* in the saliva of caries samples and believed that it may enter the salivary flow from dislodged plaque or carious substances (Jagathrakshakan et al., 2015). Moreover, *Rothia* was reportedly isolated from the saliva of children with dental caries and was investigated as a candidate biomarker for assessing the risk of caries in children (Jiang et al., 2016). However, it has also been suggested that there is a higher level of *Rothia* in caries-free individuals than in those with caries (Havsed et al., 2021). Second, *Subdoligranulum*, a member of the order *Clostridia*, is commonly found in the human intestinal flora (Chassard et al., 2014). *Subdoligranulum* deficiency was reported to be one of the main characteristics of gut dysbiosis in patients with food allergies or necrotizing enterocolitis (Abdel-Gadir et al., 2019; Lin et al., 2021). In this study, a high abundance of *Subdoligranulum* was found in the saliva of patients with WSLs, and because few previous studies have found an association between *Subdoligranulum* and the oral microenvironment, further study of this relationship is needed. Third, *Capnocytophaga* has been reported to be involved in the formation of dental plaque biofilms (Teles et al., 2012). For example, Johansson et al. reported that the composition of the supragingival plaque microbiota in Swedish adolescents with caries included mainly *Capnocytophaga* and *Actinomyces* (Johansson et al., 2016). However, Zhu et al. discovered that *Capnocytophaga* in saliva can help predict the recurrence of early childhood caries (Zhu et al., 2018). Fourth, *Olsenella* can produce lactic acid, and two recent studies using high-throughput sequencing technology also revealed that *Olsenella uli* was enriched in the saliva of caries patients (Kraatz et al., 2011; Nardello et al., 2020). Additionally, *Lachnoanaerobaculum* was abundant in the plaque of patients with caries (Lee et al., 2021), and in our study, we found that it was significantly enriched in the saliva of patients with WSLs, which may be related to its flagella and motility. Moreover, *Abiotrophia* exists in dental plaque and saliva and is one of the early colonized plaque microbial communities (Takeshita et al., 2015). It has been reported that the content of *Abiotrophia* in the plaque of adolescent patients with caries is significantly higher than that of patients without caries (Havsed et al., 2021). In addition, ElSalhy et al. found that the level of *Abiotrophia defectiva* in the saliva of children with caries was increased, and its level was found to be positively correlated with the severity of caries (ElSalhy et al., 2016). Based on the data from previous studies combined with the results of this study, the above six bacterial genera might be potential salivary biomarkers for assessing adolescents’ risk of developing WSLs during clear aligner therapy. *Subdoligranulum* and *Lachnoanaerobaculum* were first reported as differentially expressed genera in the saliva of adolescents with WSLs. This finding may be related to the fact that clear aligners alter the oral microenvironment in a way that is different from other orthodontic appliances.

Pathway enrichment analysis identified six primary metabolic pathways, most of which were related to amino acid metabolism. These results were in accordance with the study of Yang et al., who found that amino acid metabolism was the primary metabolic activity in the saliva of young adults with caries (Yang et al., 2014). Wang et al. showed that the salivary microbiome of patients treated with clear aligners was enriched in amino acid metabolism and carbohydrate metabolism (Wang et al., 2019). However, according to Liu et al., amino acid and carbohydrate metabolism were enriched in deep-dentin caries, but carbohydrate metabolism was the most abundant pathway in deep-dentin carious lesions (Liu et al., 2020). Thus, we speculate that amino acid metabolism predominates in WSLs associated with clear aligners, whereas carbohydrate metabolism seems to be instrumental in the development of caries.

The results of saliva-targeted metabolomics showed higher levels of 22 metabolites and lower levels of 5 metabolites in the WSL group than in the control group. Based on the results of the subsequent integrative analysis of multiomics data, we found that the pathogenesis of WSLs was closely related to changes in the relative concentrations of proline, glycine and lactate. Saliva contains acidic and basic proline-rich proteins (PRPs) that are associated with caries susceptibility (Zakhary et al., 2007). Pereira et al. found a clear increase in the levels of 9 amino acids, including proline and glycine, in the saliva of caries-active children (Pereira et al., 2019). Moreover, acidic PRPs can promote the attachment of certain important bacteria, such as *Actinomyces viscosus* and *Streptococcus mutans*, both of which cooperate in biofilm formation (Gibbons, 1989). Fonteles et al. found that proline was frequently absent in patients without caries, while glycine deficiency was more often observed in patients with caries (Fonteles et al., 2009). Hence, the authors suggested that the presence of proline increases the risk of developing caries, while the presence of glycine can reduce that risk. The study also pointed out that proline in saliva was mainly produced by bacterial metabolism and was not related to the presence of *Streptococcus mutans*. However, in the present study, both proline and glycine were found to be increased in the WSL group and were positively correlated with increases in three bacterial genera, namely, *Lachnoanaerobaculum*, *Rothia* and *Subdoligranulum*. The reason for this inconsistency may be due to the difference in research methods. Additionally, many factors affect amino acid changes, and the reaction pathways involved are numerous, complex, and overlapping. Furthermore, we observed lower levels of lactic acid, gamma-linolenic acid, 2-hydroxybutyric acid, valeric acid and glucose in the saliva of the WSL group than in the control group. Pereira et al. also found that lactic acid and butyrate were reduced in caries patients (Pereira et al., 2019). Lactic acid is frequently mentioned in the literature on dental caries. However, some previous studies hold a different opinion, reporting elevated lactate concentrations in patients with dental caries (Fidalgo et al., 2013), while others have come to the opposite conclusion (Pereira et al., 2019; Schulz et al., 2020). This discrepancy might be related to the good solubility of lactic acid in aqueous systems and the strong buffering capacity of saliva. We should be cautious in interpreting the lactic acid level in this study, and its relationship with WSLs needs to be further explored.

Finally, according to the integrative analysis of the microbiome and metabolome in our research, the levels of *Lachnoanaerobaculum*, *Rothia* and *Subdoligranulum* in the saliva of patients in the WSL group were related to the levels of glycine, proline, glutamine, and serine. *Lachnoanaerobaculum* has been found to produce butyric acid, acetic acid, H2S and NH3 as metabolic products (Hedberg et al., 2012). In addition, *Rothia* has been reported to reduce nitrate and transform several carbohydrates, including glucose and galactose, to yield butyric acid (Rosier et al., 2022; Yin et al., 2022). *Subdoligranulum* can also produce butyric acid (Chassard et al., 2014). Interestingly, the present study did not find a potential relationship between the three bacterial genera mentioned above and butyric acid, but an interaction between their presence and the levels of specific amino acids was observed. To the best of our knowledge, this study is the first to associate the above three bacterial genera with amino acid changes in the saliva of patients with WSLs. This finding suggested that clear aligners may affect the oral microenvironment by altering the microbiota and their associated metabolic functions.

In conclusion, when adolescents undergo long-term clear aligner therapy with poor oral hygiene habits, such as eating with clear aligners and not brushing teeth regularly, clear aligners can disrupt the balance of the oral microecosystem, leading to oral microbiota dysbiosis and increasing the risk of developing WSLs. Monitoring bacterial biomarkers and intervening in the colonization and metabolism of relevant bacterial taxa will be an effective method to prevent and treat WSLs associated with clear aligners in the future. Dentists are also advised to instruct adolescent patients to maintain oral hygiene and not to eat while wearing clear aligners whenever possible. If wearing clear aligners is a necessary clinical treatment need, dentists should pay special attention to the prevention of WSLs and be aware of treatment options. Although the use of a multiomics method to reveal links between the microbiome, metabolome and WSLs was a strength of this study, these results do not define cause-and-effect relationships. Given that the sample size in this study was relatively small, one should not overstate the implications of these findings. Future studies with larger sample sizes and confirmatory experiments are necessary to validate the findings of our study.

## Data availability statement

The datasets presented in this study can be found in online repositories. The names of the repository/repositories and accession number(s) can be found in the article/supplementary material.

## Ethics statement

The studies involving human participants were reviewed and approved by School of Stomatology, Air Force Medical University Ethical Review Board, Xi’an, China. Written informed consent to participate in this study was provided by the participants’ legal guardian/next of kin. Written informed consent was obtained from the minor(s)’ legal guardian/next of kin for the publication of any potentially identifiable images or data included in this article.

## Author contributions

ZS contributed to conception, design, collected samples, data analysis and interpretation, and drafted and critically revised the manuscript. SF contributed to conception, design, collected samples, data acquisition, and critically revised the manuscript. TG contributed to conception, design, and drafted and critically revised the manuscript. YW contributed to data interpretation and critically revised the manuscript. QL and ZJ contributed to conception, design, data acquisition and interpretation, and drafted and critically revised the manuscript. All authors contributed to the article and approved the submitted version.
